# Comparative Studies on Essential Oil and Phenolic Content with In Vitro Antioxidant, Anticholinesterase, Antimicrobial Activities of *Achillea biebersteinii* Afan. and *A. millefolium* subsp. *millefolium* Afan. L. Growing in Eastern Turkey

**DOI:** 10.3390/molecules27061956

**Published:** 2022-03-17

**Authors:** Gizem Gülsoy Toplan, Turgut Taşkın, Gökalp İşcan, Fatih Göger, Mine Kürkçüoğlu, Ayşe Civaş, Gülay Ecevit-Genç, Afife Mat, Kemal Hüsnü Can Başer

**Affiliations:** 1 Department of Pharmacognosy, Faculty of Pharmacy, Istinye University, Istanbul 34010, Turkey; 2 Department of Pharmacognosy, Faculty of Pharmacy, Marmara University, Istanbul 34668, Turkey; turguttaskin@marmara.edu.tr; 3 Department of Pharmacognosy, Faculty of Pharmacy, Anadolu University, Eskisehir 26470, Turkey; giscan@anadolu.edu.tr (G.İ.); fatihgoger@anadolu.edu.tr (F.G.); mkurkcuo@anadolu.edu.tr (M.K.); 4 Department of Pharmacy and Pharmaceutical Services, Tuzluca Vocational School, Igdır University, Igdir 76000, Turkey; ayse.civas@igdir.edu.tr; 5 Department of Pharmaceutical Botany, Faculty of Pharmacy, Istanbul University, Istanbul 34116, Turkey; gulayecevit@istanbul.edu.tr; 6 Department of Pharmacognosy, Faculty of Pharmacy, Istanbul University, Istanbul 34116, Turkey; afifemat@istanbul.edu.tr; 7 Department of Pharmacognosy, Faculty of Pharmacy, Near East University, Nicosia 99138, Cyprus; khcbaser@gmail.com

**Keywords:** *Achillea biebersteinii*, *Achillea millefolium* subsp. *millefolium*, LC-MS/MS, essential oil, GC-GC/MS, antimicrobial, antioxidant, anticholinesterase

## Abstract

The potential applications of *Achillea* species in various industries have encouraged the examination of their phytochemical components along with their biological potential. In the present study, phenolic contents and essential oil compositions together with the in vitro biological activities of the aerial parts from *Achillea biebersteinii* Afan. and *Achillea millefolium* subsp. *millefolium* Afan. collected from Turkey were evaluated. Different solvent extracts (*n*-hexane, chloroform, methanol, water) were prepared and their antimicrobial, anticholinesterase, and antioxidant activities were studied. The LC-MS/MS results revealed the presence of 16 different phenolic compounds, including chlorogenic acid, rutin, quercetin, and luteolin glycosides, in methanolic extracts. According to GC-FID and GC/MS results, the primary components of the oils were identified as 1,8-cineole (32.5%), piperitone (14.4%), and camphor (13.7%) in *A. biebersteinii* and 1,8-cineole (12.3%) and β-eudesmol (8.9%) in *A. millefolium* subsp. *millefolium*. The infusion and methanolic extracts of both species were found to be rich in their total phenolic content as well as their antioxidant and anticholinesterase activity. In contrast, the *n*-hexane and chloroform extracts of both species showed strong antimicrobial activity with MIC values ranging from 15 to 2000 μg/mL. Our findings suggest that the investigated *Achillea* species could be evaluated as potent natural agents, and further studies into the promising extracts are needed.

## 1. Introduction

Various plants have been utilized for a number of diverse reasons all over the world. In particular, aromatic plants are cultivated for use as flavoring agents in cosmetics, for food preservation, and for improving the taste of many types of food. Among aromatic plants, the Asteraceae family members have gained particular attention in the last few years thanks to their utilization in different fields [1,2]. *Achillea* is one of the valuable genera belonging to the Asteraceae (Compositae) family, widely known as “yarrow” [3]. The genus *Achillea* is represented by nearly 140 species, most of which are herbaceous perennial plants and spread across Europe, the Middle East, North and West Asia, and North America [4]. Turkey is considered as one of the main homelands of the *Achillea* species, with forty-eight species recorded in the flora of Turkey, half of which are endemic [5]. Achillea is named after the Greek hero “Achilles” [6]. For thousands of years, *Achillea* species have been utilized in different folk medicines for a variety of medical purposes, including for wound healing, hepatobiliary complaints, pneumonia, gastrointestinal disorders, and rheumatic pains [7]. Some *Achillea* species are used in the food and cosmetic industries, as well as in horticulture and as spices, drinks, and additives [3,8,9]. In Anatolian folk medicine, most *Achillea* species known as “civanperçemi” have been used to treat menstrual complaints, digestive disorders, and hemorrhoid problems and also as diuretic agents, appetizers, and wound healing agents [10].

Extensive examinations have been carried out on the phytochemical composition along with pharmacological activities of essential oils and different solvent extracts obtained from *Achillea* species [11,12,13,14,15,16,17,18]. Their antimicrobial, antioxidant, antispasmodic, and anti-inflammatory properties have been exhibited in several studies [19,20,21,22,23,24]. Regarding their phytoconstituents, a wide variety of secondary metabolites have been established in *Achillea* species, including flavonoids, essential oils, lignans, guaianolides, sesquiterpene lactone, proazulenes, alkamides, and tannins [25,26,27,28]. Due to their broad range of phytochemical compositions, these species have shown a wide spectrum of biological activities, which makes them worthwhile subjects for scientific studies. Moreover, the taxonomy of this genus is rather complex and chemical markers are helpful for identifying the species and subspecies [25,26,27,28,29]. On the other hand, the genus appears to have common intraspecific chemical variation and polymorphism, as evidenced by a recent study [24].

Free radicals, which are continuously created in the body as a result of cell metabolism, are harmful to the organism. A plethora of different diseases, including diabetes, neurodegenerative disorders, cancer, and atherosclerosis, are exacerbated as a result of this form of damage [30,31,32,33,34]. Since natural substances can be used in pharmaceutical, cosmetic, and food preparations, it is essential to explore the secondary metabolites present in medicinal plants and their biological properties [9].

Today, monographs issued by health authorities approve of the medicinal use of *Achillea millefolium*, which is considered to be an officinal medicinal plant, especially for the treatment of skin illnesses, lack of appetite, minor wounds, urinary and genital complaints, and digestive issues [1,6,7,35] Furthermore, the flowers of *Achillea millefolium* manufactured by different processes are used for lenitive, soothing, purifying, and refreshing purposes in cosmetic products and take part in plant “cosmetics monographs” prepared by the Committee of Experts on Cosmetic Products [36]. *A. millefolium* and *A. biebersteinii* are the most popular and valuable species of *Achillea*, and both have been studied by several researchers and shown to have a diverse range of chemical constituents [30,37]. Investigations into these industrially important species need to be carried out in order to better understand their chemotypes and pharmacological potential.

Within the scope of our research into traditional plants used in the eastern region of Turkey, we carried out phytochemical studies on the in vitro pharmacological properties of two *Achillea* species, which are used in both veterinary and public health by the local people of the region. As far as we know, despite the scarcity of research on the essential oil content and antimicrobial activities of these species, no comprehensive examination on the biological activity of various extracts or phenolic composition of the plants has been published. The current study is intended to contribute to knowledge of the biological potential of *Achillea* species in conjunction with their phytochemical composition.

## 2. Results and Discussion

### 2.1. Sample Preparation

The hydrodistillation method was used to obtain the essential oil of the aerial parts of *A. biebersteinii* and *A. millefolium* subsp. millefolium with 0.5% and 0.2% yields, respectively. The solvent extract yields of the samples are given in Table 1, where extractable compounds are expressed as (EC)/gram of dry weight (DW). The highest yields were recorded in the methanolic extracts of *A. biebersteinii* (2.803 g) and *A. millefolium* subsp. millefolium (3.64 g), while the lowest yields were measured for the *n*-hexane extract in *A. biebersteinii* (0.154 g) and the infusion of *A. millefolium* subsp. *millefolium* (0.92 g).

### 2.2. The Essential Oil Composition of Two Achillea Species

The essential oil contents of *A. biebersteinii* and *A. millefolium* subsp. millefolium were characterized by GC-FID and GC/MS analyses. According to the analyses, fifty-six and sixty-four compounds were determined, accounting for 86.1% and 86.8% of the total substances in the essential oils, respectively. The retention indices and percentage of compounds are given in Table 2.

In the essential oil of *A. biebersteinii*, 1,8-cineole (32.5%), piperitone (14.4%), and camphor (13.7%) were detected as predominant constituents. In addition, α-terpineol (2.8%), borneol (2.6%), camphene (1.9%), α-pinene (1.4%), *p*-cymene (1.6%), terpinen-4-ol (1.5%), and β-pinene (1.0%) were detected in moderate to low concentrations and the rest of detected compounds were not found to have concentrations higher than 1% in the oil studied. Additionally, decanoic, dodecanoic, and hexadecanoic acids were detected in fairly low concentrations. In previous reports, chemical variations were demonstrated in the essential oil of *A. biebersteinii* growing in different localities around the world, including Turkey [5]. Several studies have shown the predominance of oxygenated monoterpenes in *Achillea* species [23]. Esmaeili et al. (2006) investigated the composition of the essential oil from *A. biebersteinii* growing wild in Azerbaijan and reported camphor and borneol as the main constituents, followed by 1,8-cineole [44]. The major compounds in the oil from *A. biebersteinii* growing in Iran were piperitone (17.0%), camphor (12.0%), and ascaridole (37.0%) [16]. In another study on the essential oil of the species from Iran, 1,8-cineole (32.8%) was identified as a main compound, along with carvacrol (10.9%) and piperitone (7.3%) [45]. The essential oil of the species growing in Jordan was reported to contain ascaridol (36.2%) and *p*-cymene (31.6%) as the dominant constituents, followed by carvenone oxide (6.4%) and camphor (4.7%) [46]. In studies carried out by Turkish researchers, the major constituents of the oil were determined mostly to be camphor and 1,8-cineole, followed by piperitone [5]. Furthermore, α-terpinyl acetate, borneol, and *p*-cymene were reported in high to moderate concentrations in many essential oils of *A. biebersteinii*. Comparing our findings with those of earlier studies, we found that the main constituents of the oils were quite similar; however, the percentages of the various molecules varied. Among these studies, only one investigation showed the main compounds in the oil as *p*-cymene and ascaridole, without camphor and 1,8-cineole [47]. On the other hand, Sevindik et al. (2018) studied the essential oil from the aerial parts of *A. biebersteinii* and demonstrated different major groups: 1,8-cineole (20.36%) and cyclohexanone (8.39%), followed by 2-cyclohexen-1-one (5.38%) and spathulenol (4.2%) [48]. In the literature, the variety of compounds and their concentrations are attributed to the different ecological regions of this plant as well as to the existence of different chemotypes of the species [25].

In the current study, the principal compounds of essential oil from *A. millefolium* subsp. *millefolium* were determined as 1,8-cineole (12.3%) and β-eudesmol (8.9%). Additionally, α-pinene (4.3%), caryophyllene oxide (4.2%), β-pinene (2.9%), (*E*)-nerolidol (2.7%), α-terpineol (2.7%), *p*-cymene (2.4%), terpinen-4-ol (2.2%), β-caryophyllene (1.8%), sabinene (1.8%), *cis*-chrysanthenyl acetate (1.7%), and camphor (1.3%) were identified in the oil. The results showed that a high proportion of the oil consists of oxygenated monoterpenes and sesquiterpenes, which were both found in almost equal proportions. Three fatty acids were detected in the oil—namely, decanoic acid (4.6%) and hexadecanoic acid (4.7%) in moderate concentrations and dodecanoic acid (0.4%) in low concentrations.

Numerous studies have demonstrated that significant differences exist in the essential oil content of many *Achillea* species [3,49,50]. In terms of morphology, ploidy level, and chemical composition, *A. millefolium* is a highly polymorphic assemblage comprising numerous taxa [20]. In the examinations carried out so far, the main constituents were found to be 1,8-cineole, α/β-pinene, sabinene, camphor, linalool, α-terpineol, borneol, α/β-thujone, caryophyllene oxide, and chamazulene [3]. *A. millefolium* is considered as an officinal plant which appears in many monographs and pharmacopeia. According to the European Pharmacopoeia, *A. millefolium* should contain proazulenes in the essential oil. Although azulene was detected in the essential oil of some species grown in Europe, it was not found in species growing in Turkey [5]. Orav et al. studied (2006) the EO content of *A. millefolium* collected from Estonia and revealed the major constituents to be 1,8-cineole, chamazulene, β-pinene, (*E*)-β-caryophyllene, sabinene, and germacrene D. According to their results, all the studied oils from Estonian samples showed similar chemotypes and met EP requirements in terms of their oil content [51]. In another study, volatile extracts of *A. millefolium* growing in coastal regions of Italy and Portugal were examined by Falconieri et al. (2011); α-asarone, β-bisabolene, and α-pinene were identified as major components in Italian samples, whilst *trans*-thujone, β-pinene, and *trans*-chrysanthenyl acetate were determined as major compounds in Portuguese samples [13]. Three chemotypes of the essential oils of *A. millefolium* growing in Serbia were reported to contain (1) β-pinene, *trans*-caryophyllene, and chamazulene; (2) lavandulyl acetate, trans-caryophyllene, and chamazulene; and (3) germacrene D, *trans*-chrysanthenyl acetate, and *trans*-caryophyllene [5,52]. The essential oil of *A. millefolium* growing in Sivas (in the eastern part of Turkey) was found to be dominated by 1,8-cineole, α-terpineol, camphor, borneol, and β-pinene [53]. The essential oils were characterized by α-bisabolol, caryophyllene oxide, and muurolo-4,10(14)-dien-1-ol in Yozgat samples [18]. The aerial parts of *A. millefolium* collected from Elazığ (southeast part of Turkey) and Ardahan (northeast part of Turkey) were investigated in terms of their oil content; the major components were revealed to be δ-cadinene, limonene oxide, caryophyllene oxide allo-aromadendrene, and β-caryophyllene in the Elazığ sample, while the major compounds were found to be 1,8-cineole, α/β-pinene, and terpinen-4-ol in the Ardahan sample [49,54]. When comparing all these studies conducted on the essential oil compositions of several *A. millefolium* with our present work, differences were observed in terms of the major components and their amounts. To sum up all the investigations, differences in the chemical compositions of the essential oils of *Achillea* species can be seen despite their being collected from similar regions. The polymorphic variants and growth conditions of these species, such as geographical region, altitude, climate, as well as the vegetation season, are assumed to be responsible for the differences in the components and their amounts.

### 2.3. The Results of LC–MS/MS Analysis

**Figure 1:**
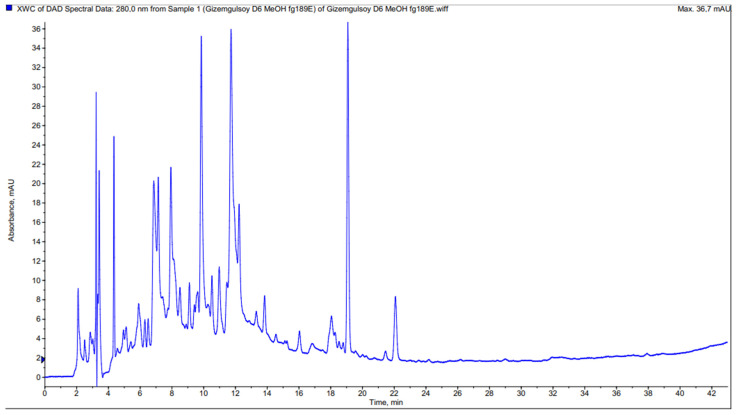
HPLC chromatogram of methanolic extract from *A. biebersteinii*.

**Figure 2:**
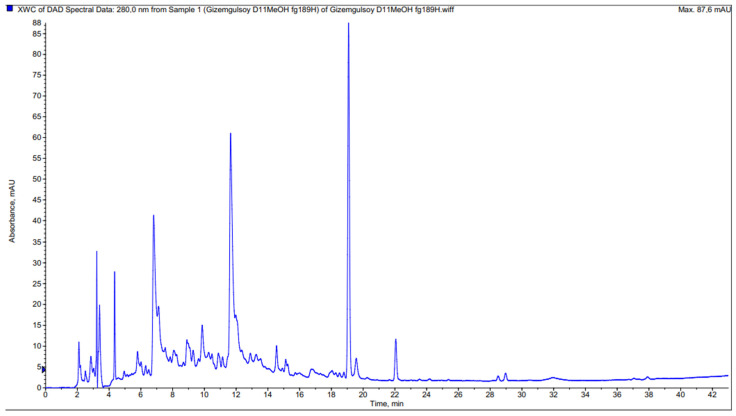
HPLC chromatogram of methanolic extract from *A. millefolium* subsp. *millefolium*.

The phenolic contents of methanol extracts prepared from the aerial parts of two *Achillea* species were determined by LC–MS/MS. Sixteen different phenolics were identified and demonstrated with UVmax spectra, retention time, and all MS data for each compound in Table 3. The chromatograms are presented in Figure 1 and Figure 2.

Compound **1**, which showed a pseudomolecular ion at *m*/*z* 191 with a product ion at *m*/*z* 173, was identified as quinic acid. Compound **3** contained a molecular ion peak at *m*/*z* 353 [M − H], which was fragmented to quinic acid (*m*/*z* 191) formed after the loss of a caffeoyl unit (−162 amu). Therefore, compound **3** was identified as a caffeoylquinic acid (chlorogenic acid) derivative. Compound **12** presented a molecular ion peak at *m*/*z* 515 [M − H], which was fragmented to chlorogenic acid *m*/*z* 353 [M − H]^−^ due to the loss of a caffeoyl moiety. After the loss of a two-caffeoyl unit (−162 amu) from the molecular ion peak, a quinic acid ion peak was observed at *m*/*z* 191; thus, compound **12** was identified as dicaffeoylquinic acid.

Compound **2** contained a pseudomolecular ion at *m*/*z* 133 with a product ion at *m*/*z* 115 due to the loss of H_2_O. The fragmentation behavior of compound **2** matched that of malic acid, so compound **2** was identified as malic acid.

Compound **4** contained a molecular ion peak at *m*/*z* 315 [M − H], which was fragmented to ion at *m*/*z* 153 due to the loss of a hexose moiety. According to a literature search, this fragmentation behavior matched that of protocatechuic acid hexoside.

Compounds **6**, **7**, and **10** showed an aglycon ion at *m*/*z* 301 (quercetin). Compound **6** had a 162 amu (hexose moiety) higher molecular weight than that of quercetin, so compound **6** was identified as quercetin glucoside; meanwhile, compound **7** had a 309 amu higher molecular weight, which indicated a rutinose moiety characterizing the compound as quercetin rutinoside. Compound **10** was identified as quercetin glucuronide according to the loss of a glucuronic acid moiety (−176) from the molecular ion peak.

Compound **15** was identified as luteolin with a molecular ion peak at *m*/*z* 285 which fragmented to ion at *m*/*z* 133. Compound **8** was identified as luteolin glucoside and had a 162 amu higher molecular weight than compound **15**. The identifications of compound **14** (isorhamnetin) and compound **11** (isorhamnetin glucoside) were also performed similarly.

In summary, the LC–MS/MS results indicated the presence of malic acid, quinic acid, chlorogenic acid derivative, protocatechuic acid hexoside, myricetin glucoside, quercetin glucoside, luteolin glucoside, apigenin glucoside, methylmyricetin glucoside, quercetin glucuronide, isorhamnetin glucoside, dicaffeoylquinic acid, and isorhamnetin in the methanol extract of *A. biebersteinii*. Furthermore, chlorogenic acid derivative, malic acid, quinic acid, protocatechuic acid hexoside, luteolin glucoside, quercetin glucoside, quercetin rutinoside, apigenin glucoside, and luteolin were identified in the methanolic extract of *A. millefolium* subsp. *millefolium*. Phenolic compounds were identified by the comparison of their retention indices and mass spectral references. Both samples contained apigenin, quercetin, and luteolin glucosides as well as chlorogenic acid derivatives. Interestingly, dicaffeoylquinic acid, one of the common phenolics in many *Achillea* species, was not detected in the methanolic extract of *A. millefolium* subsp. *millefolium*. Our findings showed similarity with previous reports on the phenolic components of several *Achillea* species. Zengin et al. (2017) described the phenolic acids in methanol, water, and ethyl acetate extracts of *A. biebersteinii* and *A. millefolium* subsp. *millefolium* collected from different parts of central Anatolia (Afyon), Turkey. In their study, the presence of 3-caffeoylquinic acid (CQA), 4-CQA, protocatechuic acid, caffeic acid, 1-feruloylquinic acid (FQA), 1,3-diCQA, 3,4,5-triCQA, 3,5-diCQA, 4-FQA, and 3,4-diCQA was demonstrated [30]. However, several LC/MS studies conducted on *Achillea* species showed that flavonoids besides phenolic acids were also present in the extracts. According to the results of these studies, the most abundant flavonoids were apigenin, luteolin, and quercetin, along with their mono- and diglycosides in the *Achillea* genus. A review of the related literature shows that *Achillea* species contain hydroxycinnamic acids rather than hydroxybenzoic acids, with chlorogenic and caffeic acids being the most commonly reported hydroxycinnamic acids. There are several variables that might affect the phenolic content of a sample, including the solvents present; the extraction processes used; and other plant species-related characteristics, such as the plant’s age, genetics, geographic location, and harvesting season.

### 2.4. Determination of Antioxidant Activities from Two Achillea Samples

In food, cosmetics, and biological systems, oxidation is responsible for a wide range of negative consequences for human health, as well as for the stability and preservation of food and pharmaceutical products [34]. As antioxidants are important for avoiding or delaying the development of oxidative stress, they have received a great deal of interest as culinary preservatives, natural health products, and food supplements [58,59,60]. The secondary metabolites of plants, particularly essential oils and phenolic compounds, have been shown in several studies to reduce oxidative damage and prevent free radicals from causing cellular damage [34].

It is recommended to exploit numerous assays to evaluate the antioxidant property of plant-based materials, not only to better understand the action of different pathways but also to provide a more comprehensive analysis of their antioxidant capacity [60]. As a consequence, three different in vitro methods were performed to test the antioxidant capabilities of the extracts obtained from two *Achillea* species in the present study.

The 2,2-diphenyl-1-picrylhydrazyl (DPPH) method, ferric reducing ability (FRAP), and cupric reducing antioxidant capacity (CUPRAC) assays are tests that are commonly used for determining a compound’s potential to act as a free radical scavenger or hydrogen donor, as well as for determining the antioxidant activity of medicinal plants and foods [60,61,62]. In the present study, these techniques were used to assess the antioxidant potential of the different extracts from the plants. Table 1 summarizes our findings.

The methanol (59.7 mg AaE/g) extract and water (55.3 mg AaE/g) extracts of *A. biebersteinii* exhibited the highest DPPH radical scavenging activity, while no significant differences were detected in the DPPH radical scavenging abilities of the other extracts. The DPPH radical scavenging ability of the methanol and water extracts of *A. millefolium* subsp. *Millefolium* was found to be higher than that of the chloroform extract. Furthermore, it was observed that the plant’s *n*-hexane extract had no DPPH radical scavenging action. Both methanol extracts and infusion from *A. biebersteinii* and *A. millefolium* subsp. *Millefolium* were shown to have antioxidant activities that were fairly similar. Furthermore, the plant extract’s antioxidant properties were assessed based on their capacity to decrease the TPTZ-Fe (III) complex to TPTZ-Fe (II). In comparison to the other extracts, the FRAP value of the methanol extracts of the *A. biebersteinii* (0.321 mM Fe^2+^/mg extract) and *A. millefolium* subsp. *millefolium* (0.373 mM Fe^2+^/mg extract) had the highest value. The ferric reducing ability of all extracts from both plants was lower than that of the BHT compound (1.1 mM Fe^2+^/mg). The CUPRAC assay was used to measure the cupric ion reduction antioxidant capability of several extracts, while the methanol extracts of *A. biebersteinii* (0.098 mMtrolox/mg extract) and *A. millefolium* subsp. *millefolium* (0.096 mMtrolox/mg extract) had the highest CUPRAC value. Furthermore, the Cuprac values of the methanol extracts and infusions derived from both plants were found to be similar. The cupric ion reduction antioxidant capability of all extracts from both plants was lower than that of the BHA compound (1.622 mM Fe^2+^/mg).

The total phenolic content of *A. biebersteinii* extracts was found to be between 9.2 and 38.8 mg GAE/g extract. Methanol extract had the highest phenolic content (*p* < 0.05), followed by water, chloroform, and *n*-hexane extracts in that order. The phenolic content of the infusion (39.1 mg GAE/g extract) and methanol (29.2 mg GAE/g extract) extracts from the *A. millefolium* subsp. Millefolium was found to be substantially (*p* < 0.05) higher than that of the other extracts.

A vast number of studies have investigated the antioxidant benefits of several *Achillea* species. A previous study reported that the total phenolic content of methanol extract from *A. millefolium* in Turkey-Ordu was 53.11 mg GAE/g dry weight, which was higher than that of the methanol extract studied in our research. Meanwhile, the ferric reducing activity of the methanol extract from this species was 258.66 μM BHAE/g dry weight, which was lower than that of the methanol extract used in our research [63]. Barış et al. (2006) investigated the total phenolic contents and antioxidant properties of the methanol extract of *A. biebersteinii* collected from Erzurum, Turkey [64]. When the results obtained were compared with the results of our study, it was determined that it contained a lower phenolic content (5.1 µg GAE/mg extract) and, parallel to this, exhibited a weak antioxidant activity [56]. It was thought that the reason for this difference might be due to the location of the plant, the different ecological conditions, and the harvest time. It is generally known that extracts prepared with different solvents may have varying polarities and, as a result, may exhibit a range of biological activities due to the presence of diverse secondary metabolites. The greater antioxidant activity of the polar extracts can be attributed to the fact that they contain a higher concentration of phenolic compounds.

### 2.5. Determination of Anticholinesterase Activities of Two Achillea Samples

With an aging population, the prevalence of neurodegenerative disorders has increased throughout the world. One of the most prevalent neurodegenerative disorders is Alzheimer’s Disease (AD), which is characterized by changes in thoughts and abnormal actions. Currently, it is known that there are approximately 40 million Alzheimer’s patients worldwide, and it is estimated that this number will reach 115 million in 2050 [65,66,67].

In the present study, the acetylcholinesterase enzyme inhibition activities of different extracts obtained from plants were examined at a 500 µg/mL concentration according to the Ellman method. The findings showed that infusion extracts were obtained from *A. biebersteinii* (94.349%) and that *A. millefolium* subsp. *millefolium* (84.254%) had significant (*p* < 0.05) enzyme inhibition activity. Notwithstanding, it was observed that the *n*-hexane and chloroform extracts obtained from both plants did not have acetylcholinesterase enzyme inhibition potential. In addition, all the extracts were found to have lower enzyme inhibition abilities than those of the galantamine compound (96.54%). On the other hand, the water extract of *A. biebersteinii* exhibited almost equivalent inhibitory effects compared to galantamine. Barut et al. (2017) reported that the methanol extract had a lower acetylcholinesterase enzyme inhibition (IC_50_:105.05 µg/mL) than galantamine (IC_50_:17.05 µg/mL) [63]. Parallel to this, in our study the methanolic extract showed a lower enzyme inhibition activity compared to galantamine. Based on our findings, both water extracts were considered to be promising mixtures for the suppression of the acetylcholinesterase enzyme. Further research into these extracts should be conducted in order to identify the components that are associated with the activity.

### 2.6. Determination of Antimicrobial Activities of Two Achillea Samples

One of the world’s most pressing health issues is the increase in antibiotic resistance. Many different antibiotics are used in treatments today. Their widespread usage and popularity have led to a rise in the number of resistant bacterial strains, and antimicrobials have become increasingly ineffective over the past decade as a result [68,69].

To determine the antimicrobial activities of essential oil and extracts obtained from *A. biebersteinii* and *A. millefolium* subsp. *millefolium* against six bacteria and seven yeast, broth dilution methods were used. The MIC results of samples and standards can be seen in Table 4 and Table 5.

The antimicrobial tests stated that the samples showed considerable inhibitory effects on the tested yeast and pathogenic bacterial strains, with MIC values ranging from 15 to 2000 μg/mL and from 125 to 2000 μg/mL, respectively. According to the antibacterial results, *P. aeruginosa* was found to be the most resistant strain against both species, while *K. pneumoniae* showed resistance to only *A. millefolium* subsp. *millefolium* extracts. Among the studied extracts, the methanol extract of *A. biebersteinii* showed moderate antibacterial effects against *S. aureus*, with MIC values of 125 μg/mL. Furthermore, the essential oil of *A. biebersteinii* showed moderate to low inhibitory effects and was most effective on *S. typhimurium* and *K. pneumoniae* with MIC values of 250 μg/mL. In addition, the chloroform and methanol extracts of *A. millefolium* subsp. *millefolium* showed moderate inhibitory properties against *S. aureus* and *S. marcescens* along with MIC values of 250 μg/mL.

Regarding their effects on yeast, both samples were found to possess remarkable antifungal properties. Among them, the *n*-hexane and chloroform extracts of *A. biebersteinii* and *A. millefolium* demonstrated better antifungal effects, particularly on *Candida utilis* at the concentrations of 31 and 15 μg/mL, respectively. Furthermore, the *n*-hexane extract of *A. millefolium* showed strong antifungal effects against *C. tropicalis* and *C. parapsilosis*, with MIC values of 15 μg/mL. The most susceptible yeasts to the essential oil of *A. biebersteinii* were *C. parapsilosis* and *C. albicans*, with MIC values of 31 and 62.5 μg/mL, respectively. While some *Candida* species are considered to be causative agents of nosocomial infections, it has been demonstrated that in one study, *C. albicans* was responsible for urinary tract infections and *C. parapsilosis* was responsible for bloodstream infections.

Recently, the number of *Candida* infections has increased due to weakened immune systems as a result of some special treatments. It is well known that sensitivity to antifungal agents can vary from one *Candida* species to another [70]. Thus, several different types of Candida yeast were used to screen the antifungal activities of the samples in the present study. Many articles have demonstrated that several extracts and essential oils of *Achillea* species have shown promising antifungal effects despite their poor antibacterial activity [48,49,50,53]. Hence, the antimicrobial effects of *Achillea* species and their metabolites should be deeply investigated to reveal the responsible components of the activity.

## 3. Materials and Methods

### 3.1. Plant Material

The aerial parts of *A. biebersteinii* and *A. millefolium* subsp. *millefolium* were collected in Ağrı 2017, from the far-eastern region of Turkey, during the flowering stage. The voucher specimens were stored at the Herbarium of the Pharmacy Faculty of Istanbul University (ISTE No.: 116569 and 116570). The plant materials were dried at room temperature and kept in a dark place.

The essential oils (EO) were extracted from the aerial parts by hydrodistillation for three hours using a Clevenger-type apparatus. The EOs were kept at +4 °C in amber-colored vials until analysis [71].

### 3.2. Preparation of Extracts

The aerial parts of *A. biebersteinii* and *A. millefolium* subsp. *millefolium* were powdered using a laboratory-type mill and then extracted with different solvents in the order of *n*-hexane, chloroform, and methanol using a Soxhlet apparatus. After that, the extracts were run through a Whatman paper filter and dried at a temperature below 40 °C using decreased pressure and evaporation. Additionally, the maceration method was used to prepare water extracts. The powdered plant materials (10 g) were macerated by shaking using 100 mL of hot water twice and thereafter lyophilized and stored at −20 °C until analysis.

### 3.3. GC-GC/MS Analysis of Essential Oil

An Agilent 6890N GC–MSD system was used to investigate the EOs of *A. biebersteinii* and *A. millefolium* subsp. *millefolium* using capillary Gas Chromatography (GC) and Gas Chromatography-Mass Chromatography (GC/MS). The GC/MS analysis was performed on an Agilent 5975 GC/MSD instrument (Agilent, USA; SEM Ltd., Istanbul, Turkey). Using the same column and operating conditions as GC/MS, simultaneous injection was performed to create the same elution sequence. The Innowax FSC column (HP, SEM Ltd., Istanbul, Turkey) (60 m 0.25 mm; film thickness 0.25 μm) was utilized in the experiment, and the FID temperature was adjusted to 300 °C. The carrier gas used was helium (0.8 mL/min). The temperature of the GC oven was maintained at 60 °C for ten minutes before being increased to 220 °C at a rate of 4 °C/min, then held at 220 °C for ten minutes before being set to 240 °C at 1 °C/min. The split ratio was adjusted 40:1. The temperature of the injector was 250 °C. At 70 eV, mass spectra were collected. The mass range was 35–450 *m*/*z*. The Adams Library, the Baser Library of Essential Oil Constituents, the Wiley GC/MS Library, and the MassFinder Library were used to compare the mass spectra of the EO constituents. 

### 3.4. Determination of Phenolics Using LC-MS/MS

A Shimadzu HPLC 20A system (Shimadzu, Tokyo, Japan) was used in conjunction with an Applied Biosystems Q-Trap 3200 LC-MS/MS (3200 Q TRAP. Mundelein, IL, USA) system to identify phenolic chemicals. At a mass range of 150–800 amu, mass spectrum studies were conducted in the negative ionization mode. For the chromatographic analysis, a 250 × 4.6 mm, 5 µm ODS analytical column was employed at 40 °C. UV Chromatograms were taken at 280 and 320 nm. CH_3_OH:H_2_O:CH_2_O_2_ (10:89:1, *v*/*v*/*v*) (solvent A) and CH_3_OH:H_2_O:CH_2_O_2_ (89:10:1, *v*/*v*/*v*) (solvent B) were used for the gradient analysis at a flow rate of 1 mL/min. The content of B was increased from 15% to 100% over 40 min.

### 3.5. Determination of Total Phenolics from Samples

The total phenolic content of four separate extracts from the aerial parts of plants was determined using the Folin–Ciocalteau reagent according to the method described by Slinkard and Singleton (1977) with slight modifications [72]. In a nutshell, 5 µL of extract (5–0.5 mg/mL) and 225 µL of water were combined in a tube. The mixture was then mixed with 5 µL of Folin–Ciocalteu reagent (diluted 1/3 with distilled water) and 15 µL of 2 % sodium carbonate solution. After that, the mixture was let to rest for two hours at room temperature before the absorbance at 760 nm was measured against a standard reference. The extracts’ total phenolic content was measured in milligrams of gallic acid equivalents per gram of extract

### 3.6. Determination of Antioxidant Activities

CUPRAC, DPPH•, and FRAP tests were used to determine the antioxidant capacity of the extracts.

The antioxidant capacity of the samples was determined according to the method described by Apak et al. (2004), with slight modifications [73]. On a plate, 1 mL of Cu (II) (10 mM), neocuproine ethanolic solution (7.5 mM), and 1 M NH4Ac buffer solution were combined. A total of 1 mL extract and 0.1 mL pure EtOH were added to the starting mixture to leave the final quantity of 4.1 mL. The solution’s absorbance at 450 nm was measured after ten seconds of vortexing and compared to a reagent blank. CUPRAC measurement samples were shown to be Trolox equivalents (mM Trolox/mg extract).

The free radical scavenging ability in four different extracts was tested using the method of Fu et al. (2010), with slight modifications [74]. To summarize, 240 µL of DPPH• solution (0.1 mM) was combined with 10 µL of extracts (5 mg/mL–0.5 mg/mL) at various concentrations. The combination was then held at room temperature for another 30 min before being used. Using a microplate reader set at 517 nm, the absorbance of the mixture was measured in comparison to a standard. The experiment was repeated three times, with the results given as mg AaE/g extract.

The reducing power of each extract was measured according to the method described by Benzie and Strain (1996), with slight modifications [75]. In a nutshell, the FRAP reagent (3.8 mL) was combined with samples (0.2 mL) and the absorbance of the mixture was evaluated 4 min later in comparison to a standard at 593 nm. The FRAP values of the samples were represented as mM Fe^2+^/mg extract in a standard curve, which was produced using FeSO_4_.

### 3.7. Anticholinesterase Activity of the Samples

The inhibition of the cholinesterase enzymes in the samples was measured with various modifications using a 96-well microplate reader developed by Ellman et al. (1961). To begin with, all reagent solutions were prepared in 50 mM of Tris-HCl buffer (pH 8.0). (daily). The AChE solution and each sample were then combined with 40 μL of Tris-HCl buffer at a 20 μL. For 10 min, this combined solution was allowed to stand at 25 °C. The reaction was then begun by adding 20 μL of ATChI (50 mM) to the mixture and incubating the whole solution for 5 min at room temperature. The reaction mixture was then combined with 100 μL of DTNB (20 mM including 1M NaCl and 0.2 M MgCl_2_.6H_2_O). and its absorbance at 412 nm was compared to a reference. Each experiment was carried out three times in total. Galantamine was used as the control substance [76].

### 3.8. Antimicrobial Activities of the Samples

Anticandidal and antibacterial tests were performed according to partly modified CLSI M27-A2 and M7-A7 reference protocols. Amphotericin-B and Ketoconazole (Sigma-Aldrich, St. Louis, MO, USA) were used as standard antifungal agents, while Chloramphenicol and Ampicillin (Sigma-Aldrich) were used as antibacterials. *Candida albicans* ATCC 10231, *Candida utilis* NRRL Y-900, *Candida tropicalis* NRRL Y-12968, *Candida albicans* ATCC 90028, *Candida tropicalis* ATCC 750, *Candida parapsilosis* ATCC 22019, and *Candida krusei* ATCC 6258 were used as test strains for an anticandidal assay. *Escherichia coli* NRRL B-3008, *Staphylococcus aureus* ATCC 6538, *Pseudomonas aeruginosa* ATCC 7853, *Salmonella typhimurium* ATCC 13311, *Serratia marcescens* NRRL B-2544, and *Klebsiella pneumonia* NCTC 9633 were used for an antibacterial susceptibility test.

Different from the standard protocol, samples from both *Achillea* species were diluted between the concentrations of 2 mg/mL and 0.004 mg/mL, whereas the standard antifungals were diluted following CLSI methods [77,78]. To ensure purity, stored yeast strains were resuspended on potato dextrose agar (PDA, Fluka, Buchs, Switzerland) and bacteria were inoculated onto Mueller Hinton Agar (MHA, Fluka, Buchs, Switzerland). All tests were carried out using sterile 96 U-shaped multi-well plates (Brand). Antimicrobial test results were screened after the incubation period at 35 ± 2 °C, 16–20 h. The MIC (minimal inhibitory concertation) is defined as the lowest concentration in which an optically clear well can be observed. Furthermore, according to the M27-A2 method, the recommended MIC limits of two quality control strains (*C. krusei* (ATCC^®^ 6258) and *C. parapsilosis* (ATCC^®^ 22019)) against Amphotericin-B and Ketoconazole were considered for the precision and accuracy of the assay.

### 3.9. Statistical Evaluation

The results are presented as the mean standard deviations (SD) of three individual parallel investigations. After running ANOVA testing, a Tukey Multiple Comparison test was used to identify significant differences between means.

## 4. Conclusions

The phytochemical compositions of the essential oils and methanol extracts of *A. biebersteinii* and *A. millefolium* subsp. *millefolium* collected from the far-eastern part of Turkey were assessed to determine their biological effectiveness. A comparative evaluation with previous research was performed to better understand the chemical characterization and contribute to our knowledge of the chemotaxonomy of the plants. Since azulenes are an important group in *Achillea,* it is worth studying other species which grow wild throughout Anatolia. Furthermore, water and methanol extracts were found to possess stronger antioxidant properties, possibly linked to their high polyphenolic content. Additionally, both samples contained dicaffeoylquinic acid and luteolin and chlorogenic acid derivatives, which exhibit strong antioxidant effects. However, the relevance of a strong antioxidant effect may not only be due to the existence of these compounds but also to the occurrence of a possible synergistic effect with other phenolic substances. All of the samples tested were shown to have antifungal activity, while *n*-hexane and chloroform from both species in particular were found to be more effective. As a result of our findings, we believe that more extensive future research is required to determine the bioactive components of these substances and demonstrate their bioavailability.

## Figures and Tables

**Table 1:** Extract yield, total phenolic contents, antioxidant properties, and enzyme inhibition potential of the samples.

Samples	Yield ^a^	DPPH ^b^	CUPRAC ^c^	FRAP Assay ^d^	TPC ^e^	AChE Inh% ^f^
AB-*n*-hexane	0.154	3.0 ± 0.2 ^a^	0.066 ± 0.002 *	0.004 ± 0.002 *	9.2 ± 2.5 ^a^	NA
AB-Chloroform	0.166	6.9 ± 0.3 ^b^	0.083 ± 0.004 *	0.038 ± 0.004 *	22.1 ± 2.3 ^b^	NA
AB-Methanol	2.803	59.7 ± 2.5 ^c^	0.098 ± 0.003 *	0.321 ± 0.010 *	38.8 ± 1.9 ^c^	73.460 ± 0.900 *
AB-Water	1.44	55.3 ± 0.7 ^d^	0.097 ± 0.001 *	0.304 ± 0.008 *	34.4 ± 1.3 ^d^	94.349 ± 0.220 *
AMM-*n*-hexane	0.443	NA	0.046 ± 0.008 *	0.031 ± 0.007 *	13.2 ± 0.7 ^e^	NA
AMM-Chloroform	0.433	2.6 ± 0.9 ^e^	0.093 ± 0.001 *	0.067 ± 0.008 *	24.1 ± 0.6 ^f^	NA
AMM-Methanol	3.64	59.2 ± 0.4 ^f^	0.096 ± 0.002 *	0.373 ± 0.022 *	29.2 ± 1.4 ^g^	64.762 ± 0.830 *
AMM-Water	0.92	56.8 ± 0.7 ^g^	0.084 ± 0.001 *	0.098 ± 0.013 *	39.1 ± 0.9 ^h^	84.254 ± 1.268 *
BHT				1.1 ± 0.12		
BHA			1.622 ± 0.12			
Galantamine						96.54 ± 0.09

AB: *A. biebersteinii*; AMM: *A. millefolium* subsp. Millefolium. Values are reported as mean ± SD. NA: no activity; ^a^: EC/gram of dry weight (DW); ^b^: mg AaE/g extract; ^c^: mMtrolox/mg extract; ^d^: mM Fe^2+^/mg extract; ^e^: mg GAE/g extract; ^f^: % (500 µg/mL); AaE: ascorbic acid equivalent; GAE: gallic acid equivalent TPC: total phenolic content; BHT: butylhydroxytoluene; BHA: butylated hydroxyanisole; * *p* < 0.05 compared with the positive control. Different letters ^(a–h)^ in the same column indicate significant differences in the plant extract (*p* < 0.05).

**Table 2:** The essential oil composition of the aerial parts of *A. biebersteinii* and *A. millefolium* subsp. *Millefolium*.

RRI_a_	RRI_b_	Compounds	AB%	AMM%	IM
1032	1025_c_	α-Pinene	1.4	4.3	t_R_, MS
1035	1027_c_	α-Thujene	0.1	0.3	MS
1043	1036_c_	Santolinatriene	0.6	-	MS
1076	1069_c_	Camphene	1.9	0.5	t_R_, MS
1118	1110_c_	*β*-Pinene	1.0	2.9	t_R_, MS
1132	1122_c_	Sabinene	0.3	1.8	t_R_, MS
1138	1122_c_	Thuja-2,4 (10)-dien	-	0.1	MS
1176	1168_c_	α-Phellandrene	0.1	-	t_R_, MS
1188	1178_c_	α-Terpinene	0.2	0.4	t_R_, MS
1195	1193_c_	Dehydro-1,8-cineole	tr	-	t_R_, MS
1203	1198_c_	Limonene	0.3	0.4	t_R_, MS
1213	1211_c_	1,8-Cineole	32.5	12.3	t_R_, MS
1234	1234_d,h_	Isochrysanthenone	tr	-	MS
1255	1245_c_	γ-Terpinene	0.4	1.1	t_R_, MS
1280	1270_c_	*p*-Cymene	1.6	2.4	t_R_, MS
1290	1282_c_	Terpinolene	0.1	0.3	t_R_, MS
1403	1395_c_	Yomogi alcohol	0.5	-	MS
1452	1444_c_	1-Octen-3-ol	-	0.1	t_R_, MS
1497	1491_c_	α-Copaene	-	0.2	MS
1499	1496_c_	Campholenal	0.2	-	MS
1516	1510_c_	Artemisia alcohol	0.1	-	MS
1529		α-Bourbonene	-	tr	MS
1532	1515_c_	Camphor	13.7	1.3	t_R_, MS
1538	1538_h_	*trans*-Chrysanthenyl acetate	-	0.3	MS
1544	1547_g_	Dihydroachillene	0.1	-	MS
1553	1543_c_	Linalool	0.4	0.5	t_R_, MS
1556		1-Nonen-3-ol	-	0.1	MS
1571	1571_d_	*trans-p*-Menth-2-en-1-ol	0.8	0.1	MS
1583	1561_c_, 1582_e_	*cis*-Chrysanthenyl acetate	0.1	1.7	MS
1586	1576_c_	Pinocarvone	0.2	0.4	MS
1590	1579_c_	Bornyl acetate	0.5	0.5	t_R_, MS
1611	1601_c_	Terpinen-4-ol	1.5	2.2	t_R_, MS
1612	1599_c_	*β*-Caryophyllene	0.1	1.8	t_R_, MS
1617	1603_c_	Hotrienol	tr	-	MS
1638	1614_c_	*cis-p*-Menth-2-en-1-ol	0.6	-	MS
1648	1632_c_	Myrtenal	0.2	0.4	MS
1651	1651_c_	Sabina ketone	0.2	-	MS
1664	1661_c_	*trans*-Pinocarveol	0.3	0.6	t_R_, MS
1686	1679_c_	Lavandulol	-	1.2	t_R_, MS
1687	1667_c_ 1681_d_	α-Humulene	-	0.3	t_R_, MS
1689	1689_e_	*trans*-Piperitol	0.5	-	MS
1690	1675_c_	Cryptone	tr	-	MS
1706	1694_c_	α-Terpineol	2.8	2.7	t_R_, MS
1719	1700_c_	Borneol	2.6	0.8	t_R_, MS
1722	1722_f_	Cabreuva oxide II	-	tr	MS
1726	1708_c_	Germacrene D	0.9	1.1	MS
1744	1724_c_	Phellandral	0.4	-	MS
1746	1738_d_	*p*-Mentha-1,5-dien-8-ol	0.2	0.6	MS
1747	1730_c_	Piperitone	14.4	0.6	t_R_, MS
1758	1751_c_ 1758_d_	*cis*-Piperitol	0.5	-	MS
1765	1762_c_ 1764_d_	*cis*-Chrysanthenol	-	0.5	MS
1769	1768_f_	Cabreuva oxide-IV	-	tr	MS
1772	1756_c_	δ-Cadinene	-	0.5	t_R_, MS
1776	1763_c_	γ-Cadinene	-	tr	MS
1797	1790_c_	Myrtenol	0.1	0.4	MS
1802	1784_c_	Cumin aldehyde	0.1	-	MS
1845	1826_c_	(*E*)-Anethole	-	0.7	MS
1845	1836_c_	*trans*-Carveol	0.2	-	t_R_, MS
1864	1848_c_	*p*-Cymen-8-ol	0.2	0.3	t_R_, MS
2008	1986_c_	Caryophyllene oxide	0.3	4.2	t_R_, MS
2037	2036_c_	Salvial-4(14)-en-1-one	-	tr	MS
2041	2036_c_	(*E*)-Nerolidol	-	2.7	t_R_, MS
2057		*p*-Mentha-1,4-dien-7-ol	0.5	-	MS
2061	2061_e_	β-*trans*-Bejarol	-	0.7	MS
2084	2057_c_, 2084_d_	Octanoic acid	0.5	-	MS
2104	2089_c_, 2103_d_	Guaiol	-	1.0	MS
2122	2122_e_	*cis*-Bejarol	-	0.3	MS
2123	2130_d_	Salviadienol	-	tr	MS
2131	2125_c_	Hexahydrofarnesyl acetone	tr	-	t_R_, MS
2144	2127_c_	Spathulenol	0.3	1.9	t_R_, MS
2174	2159_c_	Nonanoic acid	-	0.8	MS
2187	2176_c_	γ-Eudesmol	-	0.9	MS
2209	2187_c_	T-Muurolol	-	0.3	MS
2246	2223_c_	α-Eudesmol	-	0.5	MS
2255	2238_c_	β-Eudesmol	0.5	8.9	MS
2260	2260_e_	15-Hexadecanolide	0.2	-	MS
2286	2274_c_	Decanoic acid	tr	4.6	MS
2300	2300_e_	Tricosane	0.3	-	t_R_, MS
2316	2316_d_	Caryophylladienol I	-	1.3	MS
2323		1-Bisabolone	-	tr	MS
2324	2324_f_	Caryophylladienol II (=*caryophylla-2(12),6(13)-dien-5α-ol*)	-	2.6	MS
2353	2361_d_	Caryophyllenol I (=*Caryophylla-2(12),6-dien-5α-ol*)	-	2.4	MS
2369	2371_c_, 2384_d_	Eudesma-4(15), 7-dien-1β-ol	-	0.4	MS
2392	2392_c_, 2392_d_	Caryophyllenol II (=*Caryophylla-2(12),6-dien-5β-ol*)	-	1.5	MS
2500	2500_d_	Pentacosane	0.2	-	MS
2503	2487_c_, 2496_d_	Dodecanoic acid *(=lauric acid)*	-	0.4	t_R_, MS
2931	2913	Hexadecanoic acid (=palmitic acid)	0.4	4.7	MS
		**Monoterpene hydrocarbons**	**8.1**	**13.8**	
		**Oxygenated monoterpenes**	**73.5**	**25.17**	
		**Sesquiterpene hydrocarbons**	**1.0**	**3.9**	
		**Oxygenated sesquiterpenes**	**1.1**	**29.6**	
		**Diterpenes**	**-**	**-**	
		**Others**	**2.4**	**10.5**	
		**Identified compound**	**56**	**64**	
		**Total %**	**86.1**	**86.8**	

AB: *A. biebersteinii*; AMM: *A. millefolium* subsp. *millefolium*; RRIa: RRI relative retention indices experimentally calculated against *n*-alkanes; RRIb: RRI from the literature (c [38]; d [39]; e [40]; f [41]; g [42] h [43]) for polar column values; % calculated from FID data; tr: trace (<0.1 %); Identification Method: t_R_, identification based on comparison with co-injected with standards on a HP Innowax column; MS, identified on the basis of the computer matching of the mass spectra with those of the in-house Baser Library of Essential Oil Constituents, Adams, MassFinder, and Wiley libraries.

**Table 3:** The phenolic compositions of methanol extracts obtained from *A. biebersteinii* and *A. millefolium* subsp. *millefolium*.

RT	[M − H]^−^	MS^2^	Compound	Extract	Refs.
3.7	191	173, 127	Quinic acid	M6, M11	[55]
4.1	133	115	Malic acid	M6, M11	[55]
6.8	353	239, 191, 127	Chlorogenic acid derivative	M6, M11	[55]
7.1	315	153	Protocatechuic acid hexoside	M6, M11	[56]
8.1	479	317	Similar to myricetin glucoside	M6	
8.3	463	301	Quercetin glucoside	M6, M11	[55]
9.0	609	300	Quercetin rutinoside	M11	[55]
9.9	447	285	Luteolin glucoside	M6, M11	[55]
10.0	493	331, 315, 287, 270	Similar to methylmyricetin glucoside	M6	
11.1	477	301, 179, 151	Quercetin glucuronide	M6	[28,57]
11.5	477	314, 285, 271, 243	Isorhamnetin glucoside	M6	[55]
11.7	431	268	Apigenin glucoside	M6, M11	[55]
11.8	515	353, 191, 179, 135	Dicaffeoylquinic acid	M6	[55]
12.4	315	300, 271	Isorhamnetin	M6	[55,56]
16.8	285	133	Luteolin	M11	[55]
19.8	269	149, 117	Apigenin	M11	[56]

M6: *A. biebersteinii*; M:11 *A. millefolium* subsp. *millefolium*.

**Table 4:** Antibacterial effects of the samples (MIC, µg/mL).

Bacteria Panel	Strain No.	D6-h	D6-c	D6-m	D6-i	D6-o	D11-h	D11-c	D11-m	D11-i	St-3	St-4
*Escherichia coli*	*NRRL B-3008*	*1*	*1*	*0.5*	*1*	*0.5*	*0.5*	*0.5*	*0.5*	*1*	*2*	*1*
*Staphylococcus aureus*	*ATCC 6538*	*0.25*	*0.25*	*0.125*	*1*	*0.5*	*1*	*0.25*	*0.25*	*1*	*0.1*	*0.5*
*Pseudomonas aeruginosa*	*ATCC 27853*	*>2*	*>2*	*>2*	*2*	*1*	*>2*	*>2*	*>2*	*>2*	*64*	*32*
*Salmonella typhimurium*	*ATCC 13311*	*0.25*	*0.5*	*0.5*	*2*	*0.25*	*0.5*	*0.5*	*0.5*	*1*	*1*	*1*
*Serratia marcescens*	*NRRL B-2544*	*0.5*	*0.5*	*0.5*	*>2*	*0.5*	*1*	*0.25*	*0.25*	*1*	*32*	*8*
*Klebsiella pneumoniae*	*NCTC 9633*	*0.5*	*0.5*	*0.5*	*>2*	*0.25*	*>2*	*>2*	*>2*	*>2*	*0.5*	*2*

St-3: ampicillin; St-4: chloramphenicol; D6: samples of *A. biebersteinii*; D11: *A. millefolium* subsp. *millefolium*; h: *n*-hexane extract; c: chloroform extract; m: methanolic extract; i: infusion; o: essential oil.

**Table 5:** Anticandidal effects of the samples (MIC, µg/mL).

*Candida* Panel	Strain No.	D6-h	D6-c	D6-m	D6-i	D6-o	D11-h	D11-c	D11-m	D11-i	St-1	St-2
*C. albicans*	ATCC 10231	0.125	0.25	0.25	>2	0.25	0.062	0.125	0.25	>2	0.25	0.06
*C. albicans*	ATCC 90028	0.125	0.25	0.5	1	0.0625	0.062	0.062	0.125	1	0.5	0.03
*C. tropicalis*	NRRL Y-12968	0.125	0.125	0.25	>2	0.25	**0.015**	0.062	0.25	2	0.25	0.03
*C. tropicalis*	ATCC 750	0.0625	0.0625	0.25	1	0.125	**0.015**	0.062	0.5	>2	0.25	0.03
*C. utilis*	NRRL Y-900	**0.031**	**0.031**	0.0625	0.5	0.25	**0.015**	**0.015**	0.5	2	0.06	0.06
*C. parapsilosis*	ATCC 22019	0.0625	0.0625	0.125	0.5	0.031	**0.015**	0.031	0.25	2	0.25	0.03
*C. krusei*	ATCC 6258	0.125	0.0625	0.125	0.25	0.25	0.5	0.25	0.5	>2	0.5	0.06

St-1: amphotericin-B; St-2: ketoconazole; D6: samples of *A. biebersteinii*; D11: *A. millefolium* subsp. *millefolium;* h: *n*-hexane extract; c: chloroform extract; m: methanolic extract; i: infusion; o: essential oil.

## Sample Availability

Samples of the extracts are available from the authors.
